# Surface-Enhanced Raman Scattering and Fluorescence on Gold Nanogratings

**DOI:** 10.3390/nano10040776

**Published:** 2020-04-17

**Authors:** Yu-Chung Chang, Bo-Han Huang, Tsung-Hsien Lin

**Affiliations:** Department of Electrical Engineering, National Changhua University of Education, Changhua 500, Taiwan

**Keywords:** surface-enhanced Raman scattering (SERS), localized surface plasmon resonance (LSPR), surface plasmon polariton (SPP), surface plasmon resonance (SPR), nanograting, nanofabrication, electron beam lithography

## Abstract

Surface-enhanced Raman scattering (SERS) spectroscopy is a sensitive sensing technique. It is desirable to have an easy method to produce SERS-active substrate with reproducible and robust signals. We propose a simple method to fabricate SERS-active substrates with high structural homogeneity and signal reproducibility using electron beam (E-beam) lithography without the problematic photoresist (PR) lift-off process. The substrate was fabricated by using E-beam to define nanograting patterns on the photoresist and subsequently coat a layer of gold thin film on top of it. Efficient and stable SERS signals were observed on the substrates. In order to investigate the enhancement mechanism, we compared the signals from this substrate with those with photoresist lifted-off, which are essentially discontinuous gold stripes. While both structures showed significant grating-period-dependent fluorescence enhancement, no SERS signal was observed on the photoresist lifted-off gratings. Only transverse magnetic (TM)-polarized excitation exhibited strong enhancement, which revealed its plasmonic attribution. The fluorescence enhancement showed distinct periodic dependence for the two structures, which is due to the different enhancement mechanism. We demonstrate using this substrate for specific protein binding detection. Similar periodicity dependence was observed. Detailed theoretical and experimental studies were performed to investigate the observed phenomena. We conclude that the excitation of surface plasmon polaritons on the continuous gold thin film is essential for the stable and efficient SERS effects.

## 1. Introduction

Surface-enhanced Raman scattering (SERS) spectroscopy is a powerful analytic tool for sensitive molecular quantification. Since its discovery by Fleischmann et al. on a roughened silver surface in 1974 [1], it has attracted a lot of attention because the significantly enhanced Raman signal is very helpful for specific identification of chemical and biological molecules [2]. The greatly enhanced signal is due to the interaction between the incident light and the nanometer-sized metallic structure, which gives rise to a significant enhancement in the local field at the metal surfaces due to the excitation of surface plasmon resonance (SPR) [3]. An enormous enhancement factor (EF) up to 10^14^ was achieved and single molecule detection was demonstrated independently by Nie and Kneipp et al. in 1997 using SERS [4,5]. The research activity in the field of plasmonic enhanced spectroscopy was boosted since then in the last two decades. The high sensitivity of SERS has been exploited in many applications, such as chemistry, physical and biological sciences, environmental monitoring, and medical diagnostics, etc. [2,6,7]. Because of the promising potential of SERS-based sensing, we have witnessed exponentially increased research activities in this field [8]. For SERS-sensing to have real-world impact, it is essential to have reproducible, large-area, and cost-effective SERS-active substrates.

The keys to enhance the electromagnetic near field for SERS spectroscopy are nanoscaled surface roughness and nanostructures. Various methods have been developed to fabricate SERS substrates. The fabrication techniques can be classified into two categories. The bottom-up techniques are typically based on chemical synthesis. They are usually cost-efficient and easily accessible. For example, noble metal nanoparticle (NP) self-assembly is capable of producing highly ordered NP with various shapes for efficient SERS detection [9,10]. However, these chemical methods usually have an uncontrolled NP aggregation process, which results in poor reproducibility of the SERS signal. The substrate inhomogeneity significantly limits their applications [11]. On the other hand, the nanofabrication-based, top-down techniques, such as optical [12] and electron beam (E-beam) lithography (EBL) [13,14], focused ion beam [15], and nanoimprint lithography [15,16], can produce substrates with high structural homogeneity and SERS signal reproducibility that are most desired for modern SERS applications [17,18].

Although reproducible SERS substrates with high EF could be achieved with these sophisticated nanofabrication techniques, a lift-off process of the photoresist is often necessary to produce the nanoscale features. This process is time-consuming and challenging for large-area production. For most SERS-active substrates, the enhancement is based on localized surface plasmon resonance (LSPR) on the nanoscale features, where the electrical field is greatly enhanced [19,20]. Usually, the smaller the feature the better the enhancement is when the structural geometry is optimized. However, the failure rate of the lift-off process for such small features is much higher, which thus renders a low yield of production. For commercial applications, it is desirable to have a SERS-active substrate, which is easy to fabricate, cost-effective, and reproducible with high yield.

Here, we present a facile method to produce a reliable SERS-active substrate without the time-consuming and troublesome lift-off process. The obtained SERS signal is reproducible and robust. We use an E-beam to define highly ordered nanograting patterns on the photoresist and coated the surface with a thin layer of gold. We demonstrate highly efficient SERS signals from the substrates. The grating structure enables the excitation of surface plasmon polaritons (SPPs) to propagate on the corrugated metal surface when the Bragg condition is satisfied [21].

In order to understand the origin of the enhancement effects, we compared the signals from the above-mentioned SERS substrate with photoresist (PR) lifted-off gold nanogratings, which is essentially discontinuous gold nanostripes. SERS signal is only observed on the gratings without PR lifted-off. We observed enhanced fluorescence signals for both structures, but they exhibited distinct periodicity dependence. This implied that the enhancements might be due to dissimilar mechanisms. Efficient enhancements were only observed for transverse magnetic (TM)-polarized excitation, which revealed its plasmonic origin. We systematical investigated the periodicity dependence of the enhanced Raman scattering and fluorescence signals. The signal strength of SERS and fluorescence enhancement showed similar periodicity dependence on the substrate without PR lifted-off, which indicated that they originated from the same enhancement effect. Our simulation and theoretical treatments agreed well with the experimental results. We found that the excitation of surface plasmon polariton on the continuous gold thin film was essential for the stable and efficient SERS effects, while the fluorescence on the lifted-off substrates was due to localized surface plasmon resonance. This facile method can be readily employed to produce cost-effective, large-area, SERS-active substrates with high throughput and reproducibility for practical applications.

## 2. Materials and Methods

**Figure 1:**
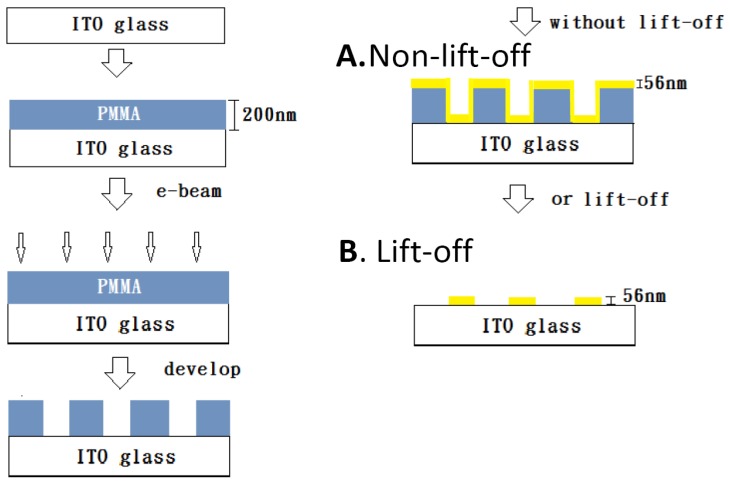
Schematic of the sample fabrication procedure. A layer of polymethylmethacrylate (PMMA) photoresist is first spin coated on the ITO glass. Nanograting patterns of the PMMA photoresist are fabricated by E-beam lithography. A layer of 56 nm gold thin film is subsequently sputtered on the nanostructure to obtain the structure **A** (nonlift-off). If the photoresist is removed by a lift-off process, we obtain the structure **B** (lift-off), which is discrete gold nanostripes.

**Figure 2:**
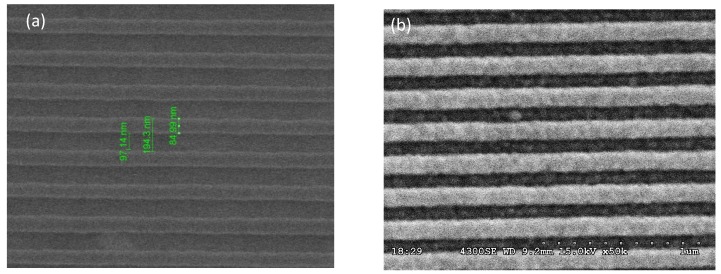
Scanning electron microscope (SEM) images of the fabricated samples. (**a**) 200 nm-period PMMA nanograting. (**b**) Gold thin film coated 200 nm-period nanograting.

The nanograting substrates were fabricated by the following procedures as illustrated in Figure 1. A layer of 200 nm-thick polymethylmethacrylate (PMMA) (Microchem A4, Kayaku Advanced Materials, Westborough, MA, USA) photoresist was spin-coated onto the ITO (indium tin oxide) glass. After prebaking to 180 °C to remove moisture and residue chemicals, we used E-beam lithography to define the nanograting patterns on the photoresist with various periods from 100 nm to 800 nm. A duty cycle of 50% was chosen for all periodicities because it had the best enhancement efficiency [22]. After the development process, we obtained periodic PMMA nanostripes as shown in Figure 2a. The grating size was 100 × 100 μm^2^ for each periodicity. In this study, we investigated SERS and fluorescence signals from two kinds of nanograting structures as shown in Figure 1: **A**. nonlift-off and **B**. lift-off. For structure **A**, the developed sample was sputtered with a layer of gold thin film on top of it without a lift-off process. The scanning electron microscope (SEM) image of a gold thin film coated 200 nm period substrate is shown in Figure 2b. For structure **B**, the photoresist was removed by a typical lift-off process, leaving periodic gold nanostripes, which were separated from each other. The thickness of the gold thin film was 56 nm for all samples as it gave the best SERS efficiency for our structure. The samples were subsequently coated with a thin layer (14 nm) of PMMA (Microchem A9) as the spacer layer to reduce the quenching effect.

For the spectroscopic studies, Rhodamin-6G (R6G) molecules (Sigma-Aldrich, St. Louis, MO, USA) were mixed in Milli-Q water (Merck, Darmstadt, Germany) to make a 10 μM solution and spin-coated on the substrate surface for subsequent measurements. Spectroscopic measurements were conducted with a confocal Raman microscope (DXR Raman, Thermo Fisher Scientific, Waltham, MA, USA). The excitation wavelength was 532 nm. The laser power was 1 mW unless mentioned otherwise. The excitation laser is linearly polarized. We rotate the samples to change the direction of excitation. For the protein-binding experiment, we add a quarter-wave plate in the optical path to make the excitation polarization circular to avoid the influence of sample orientation. The signals were collected with a 0.5NA 50× long working distance lens. The integration time was 1 s. The short measurement time prevented drying and heating of the sample. For each experimental condition, the spectra were measured at least 10 times.

In order to investigate the ability to use the substrate for biologically relevant specific binding sensing, we deposited a layer of biotinylated bovine serum albumin (BSA) on top of the gold nanogratings by immersing the sample in a 10 mg/mL BSA solution (Sigma-Aldrich, St. Louis, MO, USA) for 2 h. The BSA is sticky and easily binds to the gold surface with a layer thickness of about 3–4 nm. After raising with Milli-Q water, we immersed the samples in a 10 μM Rhodamine-conjugated streptavidin phosphate buffered saline (PBS) solution (Invitrogen, Thermo Fisher Scientific, Waltham, Massachusetts, USA) for a period of 30 min. The labeled proteins could homogenously bind to the surface of the substrates through the specific interaction between biotin and streptavidin. The total protein layer thickness was about 7–8 nm [23], which acted as the spacer layer in this case. Finally we rinsed away unbound proteins. During the microscopic measurements, the samples were maintained wet by immersing it in a thin layer of PBS solution.

In order to understand the enhancement mechanism and to verify the experimental results, we used COMSOL Multiphysics (COMSOL Inc., Stockholm, Sweden) to simulate the two kinds of nanograting structures. This numerical calculation was based on the finite element method (FEM). The calculations were performed on a single unit cell. Because the size of the grating (100 μm) was much larger than the grating period, the longitudinal length of the nanostripes could be assumed as infinitely long compared to the grating period. Hence we simplified the problem into a 2D geometry to significantly relax the required computing power and greatly reduce the calculation time. A TM-polarized plane wave (magnetic field parallel to the nanostripes) was set as the excitation source. As for material parameters used in the simulation, the complex relative permittivity of gold thin film was interpolated from ref. [24] as ε_Au_ = −5.33 + 2.55i at 532 nm. The refractive index of PMMA, ITO and water used in the simulation were 1.49, 1.88 and 1.33 respectively. In order to avoid singularities of simulation and to better model our structure, all corners are rounded by fillets in COMSOL. The corners of the PR gratings were rounded with a radius of curvature of 20 nm. The corners of gold gratings had a radius of curvature of 25 nm. For gold thin film coated PR gratings, the thickness of the gold thin film on the side walls of the PR is 15 nm, while the thickness of the gold thin film on top and in the grooves of the PR gratings was 56 nm as specified previously.

## 3. Results and Discussion

**Figure 3:**
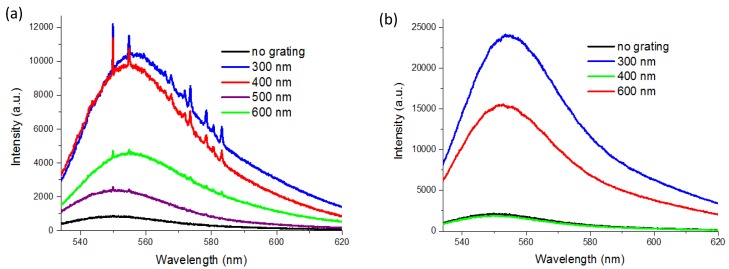
Raw data from (**a**) nonlift-off and (**b**) lift-off nanogratings of representative periods. The black curves are the spectra from substrates with no corrugated grating structure. Surface-enhanced Raman scattering (SERS) signal is only observable on the nonlift-off nanogratings. Prominent periodicity dependence of the Raman and fluorescence signals is manifested.

The measured spectra from nanogratings with various periodicities are shown in Figure 3. Figure 3a shows spectra from the nonlift-off substrates (structure A) and Figure 3b shows spectra from the lift-off substrates (structure B). Only nonlift-off nanogratings gave prominent Raman signals. As clearly seen in the figures, the signal strength is highly dependent on the grating period. For both cases, the highest enhancement on the fluorescence signals was about one order of magnitude compared to the case without corrugated grating structure (no-grating). The fluorescence signals on the nonlift-off gratings were about half of that of the lift-off samples, which should be due to fluorescence quenching as the gold coated area of the lift-off samples was exactly half of that of the nonlift-off ones. For the nonlift-off samples, the no-grating case was gold thin film coated flat PMMA, while for the lift-off samples, the no-grating case was bare ITO glass. By carefully analyzing the fluorescence signals, we noticed an obvious redshift of the spectra peak on the enhanced fluorescence. For both structures, the higher the enhancement, the more the redshift was, as was often reported in the literature of surface plasmon-enhanced fluorescence [25]. Therefore, the observed strong Raman signal could be attributed to surface plasmon-enhanced scattering (SERS).

When we compare the fluorescence signals from the two kinds of nanogratings, we found very interesting phenomena. Their fluorescence enhancement had distinct periodicity dependence. For example, although both had the highest enhancement at a period of 300 nm, for 400 nm period samples, the nonlift-off grating had a similar enhancement as the 300 nm, but the signal from the lift-off grating was even smaller than the one without a grating (control). The fluorescence intensity for the 600 nm-period lift-off grating increased again while the signal from 600 nm-period nonlift-off gratings decreased. The drastic difference intrigued us to investigate the enhancement mechanism of the two structures by the fluorescence signals. Because Rhodamine-6G (R6G) has a strong Raman signal and high quantum efficiency, we chose it and excited at 532 nm to simultaneously observe the evolution of SERS and fluorescence signals at different substrate conditions. For solely SERS applications or studies, a 785 nm laser excitation could be employed to avoid the problem of the fluorescence background. It was noted that the fluorescence intensity typically reduced rapidly in time over a few seconds immediately after the excitation radiation was switched on. This was because of the accumulation of electrons in nonradiative triplet states of the fluorophore [12,26]. However, the Raman signals were quite stable over time. Therefore, although R6G had a strong fluorescence background, it did not affect our SERS measurements.

**Figure 4:**
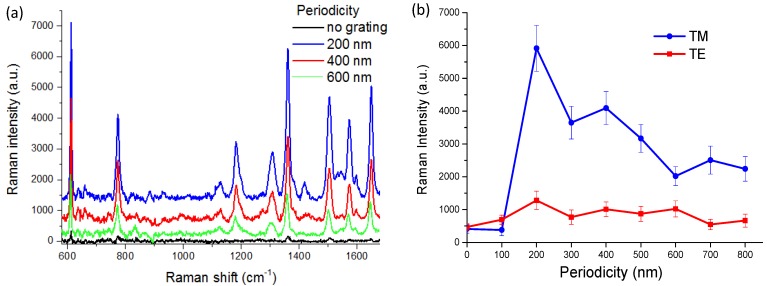
(**a**) Background corrected Raman signal from nonlift-off nanogratings. The spectra are labeled with different color respectively. The black line is the spectrum from a gratingless substrate, which is gold thin film coated flat photoresist (PR) layer (no-grating). The blue line is the spectrum from a 200 nm-period grating. Red line is the spectrum from a 400 nm-period grating. Green line is the spectrum from a 600 nm-period grating. The spectra are vertically shifted for visual clarity. (**b**) The periodicity dependence of Raman intensity for transverse magnetic (TM)- (blue circles) and transverse electric (TE)-polarized (red squares) excitation.

For our measurement conditions, no Raman signal could be found on lift-off gratings. Therefore, the following discussions about SERS are specifically for nonlift-off gratings. Figure 4a shows background subtracted SERS signals from nonlift-off nanogratings. The Raman features nicely correspond to typical R6G Raman signals [21]. We noticed that sometimes there were small Raman signals even without the corrugated grating structure. This might be due to the roughness of the PR surface. The gold thin film on flat glass substrate was checked by atomic force microscopy to have a smoothness better than 1/1000. The irregular nanoscale features might have induced enhanced local fields due to localized surface plasmon resonance [27]. However, for a short integration time of 1 s, the Raman signal on gratingless samples was often not noticeable. As will be discussed below, we attributed the greatly enhanced Raman signal to the coupling of LSPR and SPP, where SPP plays an important role in the SERS effect on the nonlift-off grating [11,28]. With the help of grating, SPP can be excited on the gold surface to propagate on the metal–dielectric interface. Figure 4b summarizes the dependence of Raman signal strength versus periodicity and excitation polarization. We noticed strong polarization dependence on the Raman signals. As is evident from the figure, the Raman signals are only moderately enhanced for TE (transverse electric)-polarized excitation and there is no obvious periodical dependence for them. On the contrary, for TM-polarized excitation (magnetic field parallel to the grating grooves), we have more than one order of magnitude enhancement for gratings of periods 200, 300 and 400 nm. Conspicuous periodicity dependence can be identified in the figure. The periodicity dependence is similar among different sets of samples. The SERS signal is reproducible and robust for samples of the same conditions. The standard deviation is less than 20% of the mean value for all cases.

**Figure 5:**
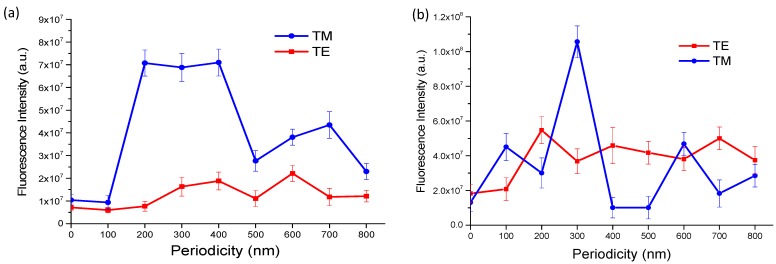
Periodicity and polarization dependence of the fluorescence intensities for (**a**) nonlift-off and (**b**) lift-off nanogratings. The blue circles and lines are for TM-polarized excitation and the red squares and lines are for TE-polarized excitation.

As mentioned previously, the different periodicity dependence of the fluorescence enhancement for the two kinds of nanograting structures is intriguing for further investigation. In Figure 5, we compare the dependence of the fluorescence signal intensity on the excitation light polarization and grating periodicity for the two kinds of nanogratings. We see a drastic difference between the two kinds of structures, which implies the enhancements might be due to distinct plasmonic effects. For both cases, only TM-polarized excitation exhibited strong enhancement at certain periodicities. The maximum enhancements compared to the gratingless substrate were both on the order of 10 times. The nonlift-off gratings had a higher enhancement factor compared to the lift-off gratings. However, the magnitude of fluorescence intensity on the nonlift-off grating was smaller than that on the lift-off grating. This was due to a higher quenching rate from the continuous gold thin film on the nonlift-off gratings, while the lift-off gratings were only 50% covered. As seen in the figure, the TE-polarized excitation also moderately increased the signal. This is because when the phase matching condition of Wood’s anomaly is satisfied, the grating couples light onto the grating [29], which increases the excitation efficiency. However, only TM-polarized excitation can excite SPP on the gold–dielectric intersurface. The excited surface plasmon can concentrate the optical field at the vicinity of the gold surface, which results in a greatly magnified electrical field. For the nonlift-off grating, the fluorescence signal was significantly enhanced for periods 200, 300, and 400 nm. As the period increased, the signal decreased and reached a minimum when the period was 500 nm. At periods of 600 nm and 700 nm, the fluorescence intensities increased again, but not as high as in the shorter period cases. This periodicity dependence is due to matching the dispersion relation of grating coupled SPP excitation as will be discussed later. However, for the lift-off gratings, as seen in Figure 5b, the signal maximum occurred at 300 and 600 nm periods. The fluorescence intensity varies with periodicity acutely. We attribute the enhancement in the case of lift-off gratings to localized surface plasmon resonances (LSPR) because the enhancement only occurred when the rigorous resonance condition was satisfied. The dispersion relation for LSPR is

(1) $$k_{sp}=\frac{2\pi }{\lambda_{sp}}=\frac{2\pi }{\lambda_{0}}\sqrt{\frac{\varepsilon_{Au}\varepsilon_{d}}{\varepsilon_{Au}+\varepsilon_{d}}}$$

where $k_{sp}$ is the surface plasmon wave vector, $\lambda_{sp}$ is the surface plasmon wavelength, $\lambda_{0}\;$ is the free space light wavelength, $\varepsilon_{Au}$ is the dielectric function of gold, and $\varepsilon_{d}$ is the dielectric function of the surrounding dielectric medium. For the excitation wavelength (*λ*_0_) of 532 nm, the resonant surface plasmon wavelength at the PMMA-gold interface is 288.3 nm. 

When the surface plasmon wavelength matches the transverse dimension of the gold nanostripe, it forms SP standing waves on the metal-dielectric interface [23]. The condition for a resonant standing SP wave across a nanostripe is

(2) $$k_{sp}\cdot \frac{\Lambda }{2}=m\pi$$

where Λ is the grating period and m is an integer. Therefore, for the lift-off grating, the first peak at 300 nm corresponds to the lowest order mode when m = 1, and the peak at 600 nm corresponds to the m = 2 order. The different excitation mechanisms might account for the drastically different periodicity dependence between the two structures.

When comparing the enhancement of Raman and fluorescence signals of the nonlift-off substrate, Figure 4b and Figure 5a, the periodicity dependence almost coincide with each other. They both had significant enhancement in a broad range of grating period from 200–400 nm, and another peak at about 700 nm. Therefore, we might infer that they were enhanced due to the same mechanism. For Raman signals, it seems the signal was larger for smaller periods and signal at the period of 200 nm was significantly enhanced. This might be due to a combined effect of SPP and LSPR coupling since the electromagnetic fields around small features are greatly enhanced by localized surface plasmons [30]. This will be discussed together with the simulation results later.

**Figure 6:**
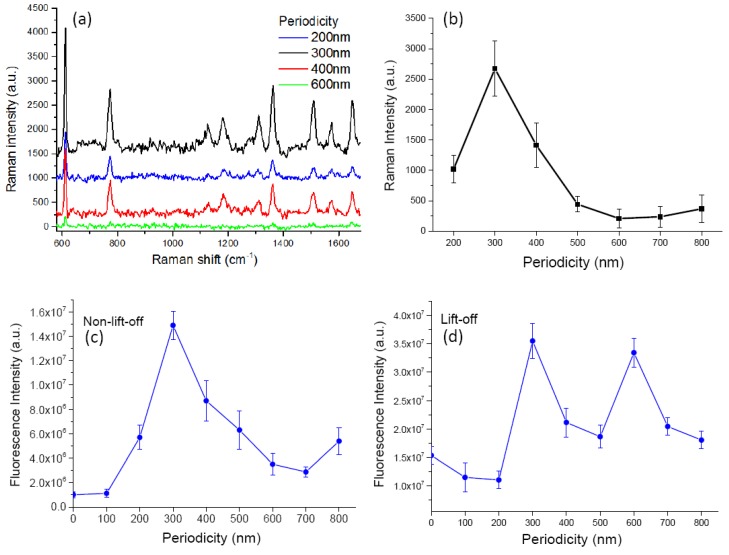
(**a**) Background corrected SERS spectra from nonlift-off nanogratings labeled with proteins. The spectra are vertically shifted for visual clarity. (**b**) The dependence of Raman intensity versus grating periodicity. (**c**) The periodicity dependence of fluorescence intensity on nonlift-off nanogratings. (**d**) The periodicity dependence of fluorescence intensity on lift-off nanogratings.

Our proposed method is capable of producing homogeneous, SERS-active substrates with reproducible and stable signals that are suitable for biological sensing. We prepared our substrate for the specific sensing of proteins using the protocol described in the Materials and Methods section. Here we used circularly polarized light for excitation to ease the sample preparation and orientation restrictions. For proof-of-principle, we exploited the specific protein binding system of biotin and streptavidin [31]. The gold nanograting surface was first deposited with a monolayer of biotinylated BSA and subsequently immersed in Rhodamine-tagged streptavidin for the interaction to take place. The measured Raman signal is shown in Figure 6a. An intense SERS signal was only observed on the nonlift-off nanogratings and the enhancement was highly dependent on the periodicity, as expected. The maximum SERS occurred at a period of 300 nm. The maximum enhancement ratio for Raman was similar to the substrates described above. However, the intensities of Raman and fluorescence signals were both smaller. This is due to quenching caused by the gold thin film. There was no 14 nm-thick PMMA spacer layer above the gold thin film. The self-adsorbed protein layer was acting as the spacer layer. Although the fluorescence intensity was smaller, the enhancement factor for SERS was the same. The signal was adequate for molecular quantification. The use of proteins as the spacer layer simplified the SERS-active substrate fabrication and was convenient for typical biochemical laboratories.

Here, we found the optimal periodicity for Raman and fluorescence was both 300 nm for nonlift gratings, which was different from the case with a PMMA spacer. Besides, in the case with a PMMA spacer on top of the gold surface, we had a second peak at a period of 700 nm. Here we had a minimum at 700 nm for both Raman and fluorescence and the second maximum seemed to occur at a longer period. This could be reasonably explained by the different SP wavelengths on the water–gold and PMMA–gold interfaces. Because there was no PMMA spacer layer, the medium above the gold thin film was water. The SP wavelength for the water–gold interface can be calculated by the dispersion relation of Equation (1). The SPP wavelength at a water–gold interface is 337.6 nm, which is longer than the PMMA–gold interface of 288.3 nm. Therefore the resonance conditions shifted to a longer period. Figure 6c,d are the periodicity dependence of fluorescence intensities for nonlift-off and lift-off nanogratings, respectively. Similarly, we see a distinct periodicity dependence between them. For the two kinds of structures, they respectively exhibit similar periodicity dependence as the case with PMMA spacer. For the nonlift-off case, it seems the resonance condition could be satisfied for a broader range of period from 200–400 nm. The enhancement decreases as the period becomes longer and increases again at about 800 nm. The same trends are seen in Raman and fluorescence signals. On the other hand, for lift-off samples, we see two peaks at periods of 300 and 600 nm. The trends are again similar to the case with a PMMA spacer on top of the grating. However, for the current condition, the 600 nm period grating exhibited almost the same enhancement as the 300 nm period grating. It seems a better resonance condition was achieved. This is reasonable if we consider the longer SP wavelength of the water–gold interface. The actual length of the interface was longer than half of the period because the gold layer had a thickness of 56 nm and the corners were usually rounded. The longer SP wavelength better fitted the resonant standing wave condition. Because of the strict resonance condition, the LSPR induced enhancement only occurred at specific periods.

**Figure 7:**
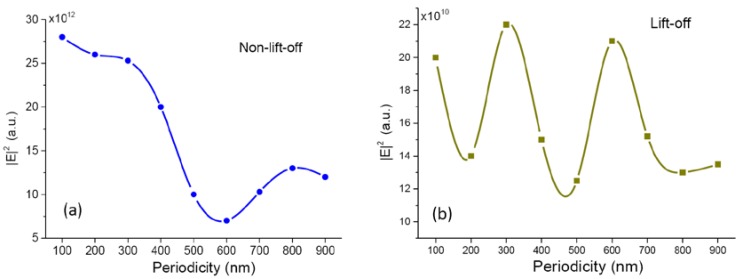
Calculated electrical field intensity on the gold surface of (**a**) non-lift-off and (**b**) lift-off nanogratings. The medium above the gold surface is water. The field strength is averaged over the entire gold surface of a unit cell.

In the above studies, we observed more than one order of magnitude enhancement of Raman and fluorescence intensities on nonlift nanogratings, while the maximum fluorescence enhancement on lift-off nanogratings was only about fivefold. To have a deeper understanding of the experimental results, we conducted numerical simulation on the two kinds of nanograting structures using the finite element method. The simulation results are shown in Figure 7. The simulated model was the structure without the PMMA spacer layer on top of the gold layer to simplify the problem and make the discussion concise. The medium above the gold layer was water. The plots show the average squared electrical fields right above the gold surface. The maximum field intensity of nonlift-off grating is about two orders of magnitude larger than that of the lift-off grating. This might account for the larger enhancement for the nonlift-off grating, because the nonlift-off grating was covered with a corrugated continuous gold thin film, which supports the excitation of propagating SPP [32]. The excitation of SPP on the gold surface was responsible for the largely enhanced field. However, the gold thin film might have quenched the generated signals, so the measured signal was not as high as simulated.

About the periodicity dependence, the simulation agrees well with the experimental results for periods longer than 300 nm. The nonlift-off grating had a minimum at a period of 600 nm as in the experimental result. The lift-off grating had maximums at 300 and 600 nm, as seen in the experimental data. The simulation confirms the LSPR-induced enhancement for the lift-off nanogratings.

For grating periods smaller than 200 nm, the field strength seems to be larger for nonlift-off structures. The electrical field for lift-off grating was also larger at 100 nm. This is because the electrical field is significantly enhanced at small features due to LSPR. However, it is difficult to make nanogratings with a period of 100 nm in real life. A period of 100 nm means the feature size is only tens of nanometers. It is challenging to make a uniform substrate with such small features. The failure rate of lift-off is also higher. As a rule of thumb, better periodicity of the grating results in a better SPP excitation and higher SERS signal intensity [33]. It was the poor homogeneity of our 100 nm-period gratings that resulted in low surface plasmon excitation efficiency. Nevertheless, as seen in the simulation results, one does not gain much field strength even with a 100-nm grating. From the viewpoint of real-world applications, it is not worth the effort to struggle with the fabrication of small gratings.

As mentioned previously, the strong field on the nonlift gratings was due to the excitation of SPP on the corrugated continuous gold thin film. The further enhancement at smaller periods was due to coupling between surface plasmon polariton and localized surface plasmons [30]. This coupling has been shown experimentally and applied for SERS sensing with a uniform gold thin layer beneath gold nanodisks and gratings [30,34]. To excite SPP on the interface, the excitation angle must satisfy the SP dispersion relation as given in Equation (3):

(3) $$k_{0}sin\theta \pm K_{G}=\frac{2\pi }{\lambda_{0}}sin\theta \pm m\frac{2\pi }{\Lambda }=k_{sp}$$

where *k*_0_ is the free space wave vector, θ is the incident angle, *K_G_* is the Bragg vector supported by the grating, and m is an integer, which refers to the order of diffraction. *k_sp_* is the surface plasmon wave vector, which is given in Equation (1).

**Figure 8:**
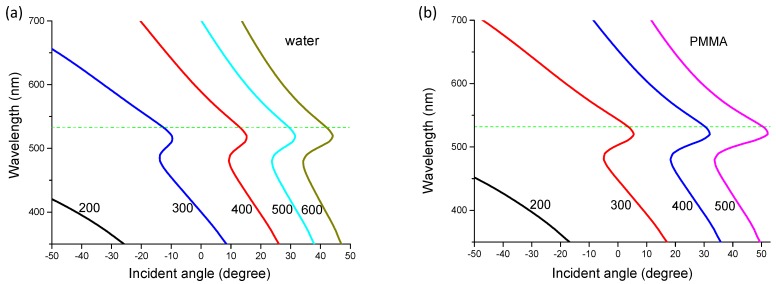
Plot of dispersion relation on (**a**) gold–water and (**b**) gold–PMMA interfaces. The numbers next to the curves are the grating period in nanometer. The green horizontal dashed line indicates the excitation wavelength of 532 nm.

Figure 8 plots the dispersion relation of Equation (3) on water–gold and PMMA–gold interfaces respectively. The NA of our objective is 0.5, which corresponds to a maximum excitation and collection angle of 30 degrees. With the large excitation angle, multiple modes of SPPs can be excited with the help of nanogratings as long as the excitation angle and grating orientation match the dispersion relation [35,36]. As seen in Figure 8a, for the water–gold interface, the resonance angle for gratings of period larger than 500 nm was larger than 30 degrees. The period of 500 nm was right on the margin of the NA, so we saw a quick drop in the excitation efficiency at 500 nm period nanograting in the simulation. In the protein-binding experiment, the difference in the signals for Raman and fluorescence at 500 nm period might be explained by the dispersion plot as well. For SERS signals, because of the lower excitation efficiency at the period of 500 nm, the Raman intensity was lower as seen in Figure 6b. However, for the fluorescence intensity, because a majority of fluorescence photons had a wavelength longer than 550 nm (emission peak of the fluorophore), their resonance occurred at an angle smaller than 30 degrees, as seen in Figure 8a. These photons may have been coupled as SPP surface waves and re-emitted at the resonance angle where the phase mating condition was satisfied [37]. The out-coupled fluorescence photons emit at a smaller angle than can be collected by the lens, thus a higher fluorescence signal was obtained.

For the PMMA–gold interface, as shown in Figure 8b, the resonance angle for 500 nm is outside the NA of the lens. Therefore we observed an intensity minimum at a period of 500 nm for the nonlift-off PMMA–coated nanograting as shown in Figure 5a. The SERS and fluorescence intensities increased again at longer periods because of the satisfaction of second order (m = 2) phase-matching conditions. For example, the second order dispersion relation of 600 nm coincided with the 300 nm period and the 700 nm coincided with 350 nm. Their resonance angles would be in the range of 0–20 degrees, which can be excited and collected with our lens. However, the efficiency of second order was smaller than the first order.

The above discussion elucidates the periodicity dependence of SERS and fluorescence intensities on the nanogratings. Because the continuous corrugated gold thin film supported the excitation of SPP, the electrical field intensity was two orders of magnitude larger on the non-lift nanograting. Due to the matching of the dispersion relation of SPP, we had the highest SERS and fluorescence signals at periods between 200–400 nm on the non-lift nanogratings. For 100 or 200 nm period grating, the dispersion relation did not directly support the excitation of SPP. However, because the corrugated small periods had Fourier components of longer periods, it could still support the excitation of SPP. The coupling of SPP and LSP at the nanosized grating grooves greatly enhanced the electromagnetic field strength [38]. Because the substrates were fabricated by E-beam lithography, our substrates had excellent homogeneity. Due to the uniform field distribution, the SERS signal was reproducible and robust. Because no lift-off process was needed, the fabrication process was easy and hassle-free. Therefore it is suitable for industrial mass production. It is also possible to make two-dimensional (2D) structures to further improve the enhancement efficiency as demonstrated in several reports [21,30,39]. For solely SERS sensing applications, a longer wavelength excitation laser of 785 nm may be employed, which resonates at a longer period. It is easier to fabricate nanogratings of a larger period. Further improvement of the enhancement factor is possible by decorating the grating surface with nanoparticles and optimizing the geometry and materials [28]. Our simple method allows the generation of SERS-active substrate in a short time with high efficiency. We envision this method to be useful as a cost-effective technique for the production of large-area, SERS-active substrates for real-world applications.

## 4. Conclusions

In conclusion, we proposed a simplified E-beam lithography method to fabricate SERS-active substrates. By eliminating the lift-off process, it is possible to make large-area, SERS-active substrates with high efficiency. The fabricated nanograting substrates were highly uniform and thus exhibited reproducible and robust SERS signals. We observed the highest SERS intensity on nanogratings of periods 200–400 nm. The enhancement had an obvious periodicity dependence. By investigating the fluorescence enhancement on the lift-off and nonlift-off nanogratings, we found the enhancement on the two structures is due to distinct plasmonic effects. The excitation of SPP was responsible for the large enhancement and SERS effect on the substrate. Our numerical simulations agreed nicely with the experimental results and indicated that further increasing of SERS efficiency is possible by the coupling of SPP and LSPR. We have demonstrated using this substrate for the detection of protein-specific binding. Our method has the potential to fabricate large-area SERS-active substrates based on the relatively matured lithographic technique in a shorter period. The proposed SERS-active substrate can be readily employed as a routine molecular sensing element for a wide range of applications, such as environmental pollutant surveillance and immunoassays.
